# Diffusion Tensor Imaging Colour Mapping Threshold for Identification of Ventilation-Induced Brain Injury after Intrauterine Inflammation in Preterm Lambs

**DOI:** 10.3389/fped.2017.00070

**Published:** 2017-04-05

**Authors:** Dhafer M. Alahmari, Beatrice Skiöld, Samantha K. Barton, Ilias Nitsos, Courtney McDonald, Suzanne L. Miller, Valerie Zahra, Robert Galinsky, Qizhu Wu, Michael John Farrell, Timothy J. Moss, Stuart B. Hooper, James T. Pearson, Graeme R. Polglase

**Affiliations:** ^1^Department of Medical Imaging and Radiation Sciences, Monash Biomedicine Discovery Institute, Monash University, Clayton, VIC, Australia; ^2^Monash Biomedical Imaging, Monash University, Clayton, VIC, Australia; ^3^Department of Women’s and Children’s Health, Karolinska Institutet, Stockholm, Sweden; ^4^The Ritchie Centre, Hudson Institute of Medical Research, Clayton, VIC, Australia; ^5^Department of Obstetrics and Gynaecology and Paediatrics, Monash University, Clayton, VIC, Australia; ^6^Department of Physiology, University of Auckland, Grafton, New Zealand; ^7^Department of Physiology, Monash University, Clayton, VIC, Australia; ^8^Department of Cardiac Physiology, National Cerebral and Cardiovascular Center, Suita, Osaka, Japan

**Keywords:** brain, delivery room, diffusion tensor imaging, magnetic resonance imaging, neonate

## Abstract

**Purpose:**

The aim of this study is to examine whether advanced magnetic resonance imaging (MRI) techniques can detect early brain injury caused by intrauterine inflammation and inappropriate initial respiratory support in preterm lambs.

**Hypothesis:**

Neuropathology caused by intrauterine inflammation is exacerbated by mechanical ventilation at birth and is detectable with advanced MRI techniques.

**Methods:**

Pregnant ewes received intra-amniotic lipopolysaccharide (LPS) 7 days prior to delivery at ~125 days of gestation (85% of gestation), whereupon lambs were delivered and randomised to receive an injurious (LPS + INJ, *n* = 6) or protective (LPS + PROT, *n* = 6) ventilation strategy. MRI of the brain was conducted 90 min after preterm delivery, using structural, diffusion tensor imaging (DTI), and magnetic resonance spectroscopy (MRS) techniques. A colour map threshold technique was utilised to compare distributions of low diffusivity voxels in the brains of LPS-exposed lambs with those not exposed to LPS (PROT, *n* = 7 PROT and INJ, *n* = 10).

**Results:**

No overt cerebral injury was identified on structural MRI images of any lamb. However, on DTI, axial diffusivity, radial diffusivity, and mean diffusivity values were lower and significantly more heterogeneous in specific brain regions of lambs in the LPS + INJ group compared to the LPS + PROT group. Colour mapping revealed lower diffusivity in the thalamus, periventricular white matter, internal capsule, and frontal white matter in the LPS + INJ group compared to LPS + PROT group. The MRS peak area ratios of lactate, relative to those for the metabolites creatine, choline, and *N*-acetylaspartate, were not different between LPS-exposed groups. Lambs exposed to LPS had lower diffusivity within the white matter regions assessed than non-LPS-treated control lambs.

**Conclusion:**

DTI colour map threshold techniques detected early brain injury in preterm lambs exposed to intrauterine inflammation and detected differences between injurious and protective ventilation strategies. DTI mapping approaches are potentially useful for early detection of subtle brain injury in premature infants.

## Introduction

Infants born preterm are at a high risk of brain injury, with the rate of neurodevelopmental disability exceeding 50%, and cerebral palsy rates at ~10% (1, 2). Further, preterm infants are born prior to adequate maturation of both breathing control and pulmonary morphology; they have a reduced respiratory surface area, a thickened air–blood gas barrier, and poor production of surfactant (3). Consequently, preterm infants often require respiratory support immediately after delivery to support lung aeration, promote pulmonary gas exchange, and facilitate the transition of the circulation from the fetal to the infant phenotype (4). However, if poorly controlled, the initiation of respiratory support can lead to brain injury by two main mechanisms: adverse fluctuations in cerebral blood flow and the initiation of a systemic inflammatory cascade resulting in an inflammatory response within the brain (5–7). Ventilation-induced brain injury can be detected within 90 min of delivery using magnetic resonance imaging (MRI) techniques, in particular, diffusion tensor imaging (DTI) (6).

Intrauterine inflammation, clinically defined as chorioamnionitis, is a common antecedent of preterm birth (8, 9) and affects ~3–10% of all pregnancies (10). We have previously demonstrated that acute (2 days) exposure to intra-amniotic (IA) lipopolysaccharide (LPS) increases the incidence and severity of ventilation-induced brain injury in preterm lambs (11). Furthermore, a protective ventilation strategy did not reduce the severity of brain injury, assessed histologically, after IA LPS (12) compared to control lambs (5). These results support clinical findings that intrauterine inflammation increases the risk and severity of postnatal white matter injury (13), periventricular leukomalacia, and periventricular/intraventricular haemorrhages (14–17). Thus, preterm infants exposed to chorioamnionitis are at a high risk of long-term neurodevelopmental impairment including cerebral palsy (13, 18, 19).

MRI is commonly used in neonatology as a diagnostic and prognostic tool and is usually conducted several days to weeks after birth (20, 21). Given that the only clinically implemented treatment for newborn brain injury, hypothermia for hypoxic–ischemic encephalopathy, needs to be initiated within the first hours after birth (22), it is critical to establish new tools for early detection of brain injury to guide current and future clinical interventions.

We have previously demonstrated the utility of DTI in detecting early brain injury using a region of interest (ROI) analysis (6). An alternative to the ROI approach is to analyse DTI data for the whole brain volume, which can detect more subtle brain injury (23). Previous studies that applied whole brain statistical analysis for DTI parameters in preterm infants demonstrated a reduction in DTI measures, thought to reflect alternations in brain microstructure (24, 25). However, this approach has not yet been applied to early detection of ventilation-induced brain injury in neonates after preterm delivery.

The objective of this study was to develop new techniques to detect early brain injury using diffusion-weighted MRI that have the potential to improve future diagnostic strategies for identifying abnormalities in white matter. In particular, we aimed to examine whether non-invasive MRI techniques can differentially detect brain injury caused by injurious or protective ventilation in preterm lambs exposed to intrauterine inflammation, factors known to cause early brain injury. Although we have demonstrated that both protective (PROT) and injurious (INJ) ventilation strategies increased brain inflammation and oxidative stress, and more so in the INJ group (5), it has not been established if DTI analyses are sensitive enough to detect the exacerbation of brain injury caused by a double hit. In our initial studies, we examined the acute response of the preterm brain to ventilation injury (5, 6). In this study, we examined the effects of ventilation injury superimposed on chronic inflammation (double hit) with MRI and magnetic resonance spectroscopy (MRS) techniques. We hypothesised that high tidal volume (V_T_) ventilation after chronic intrauterine inflammation exacerbates brain injury compared to protective ventilation and is detectable using DTI on a clinical MR scanner.

## Materials and Methods

The experimental protocol was approved by Monash Medical Centre animal ethics committee at Monash University and conducted in accordance with guidelines established by the National Health and Medical Research Council (NH&MRC) of Australia.

### LPS Treatment of the Fetus, Preterm Delivery, and Stabilisation

Ultrasound-guided IA injections of LPS (10 mg; from *Escherichia coli* 055; Sigma-Aldrich, Australia) were administered to one amniotic sac of twin-bearing ewes (Border Leicester; *n* = 12) at 118 ± 2 days of gestation (mean ± SD). Lambs were delivered by caesarean section 7 days later, dried, and a transcutaneous oxygen saturation probe was placed around a forelimb (SpO_2_; Masimo, CA, USA). The lambs were intubated and then randomised to receive either a protective ventilation strategy (LPS + PROT, *n* = 6) or injurious ventilation strategy (LPS + INJ; *n* = 6), as described previously (5). Briefly, lambs in the LPS + INJ group underwent immediate cord clamping followed by 15 min of high V_T_ ventilation (targeting 12–15 mL/kg) using a neonatal positive-pressure ventilator (Babylog 8000+, Dräger, Lübeck, Germany). After 15 min, LPS + INJ lambs received surfactant (100 mg/kg, Curosurf^R^, Chiesi Pharma, Italy), followed by tidal ventilation targeting V_T_ of 7 mL/kg. LPS + PROT lambs received prophylactic surfactant, a single 30-s sustained inflation at peak inspiration pressure (PIP) 35 cmH_2_O (Neopuff Fisher & Paykel Healthcare, Auckland, New Zealand), followed by tidal ventilation (7 mL/kg) for 3 min prior to umbilical cord clamping (26). V_T_ targeted 7 mL/kg for the protective strategy for the duration of the experiment.

### Monitoring and Care

After delivery, umbilical arterial and venous catheters were inserted for measurement of blood pressure, arterial blood gas sampling, and invasive real-time monitoring of heart rate (HR) (Powerlab: ADInstruments, Castle Hill, Australia). Anaesthesia was maintained *via* intravenous infusion of Alfaxan (10 mg/kg/h in 5% glucose, Jurox, Auckland, New Zealand). Samples of arterial blood were collected every 5 min for the initial 15 min and at 15-min intervals thereafter for measurement of the partial pressure of arterial carbon dioxide (PaCO_2_), oxygen (PaO_2_), oxygen saturation (SaO_2_), and pH (ABL30, Radiometer, Copenhagen, Denmark). Ventilation and oxygen delivery were altered to target PaCO_2_ of 45–55 mmHg and an oxygen saturation of 90–95%. Ewes were killed humanely (sodium pentobarbitone 100 mg/kg i.v.) immediately after delivery of the lambs, while still under general anaesthesia. Lambs were killed humanely (sodium pentobarbitone 100 mg/kg i.v.) after the MRI.

### Magnetic Resonance Imaging

The lambs were transferred to the MR scanner 1 h after delivery. The radiographers and team involved in supporting lambs during MRI acquisition were blinded to group assignment of each lamb. Lambs were scanned in a supine position, and ventilation was maintained using a BabyPAC portable and MR compatible ventilator (Pneupac, Smiths Medical, UK). HR and SpO_2_ were continuously monitored to guide anaesthesia maintenance, and blood gas parameters were recorded at 15-min intervals to monitor lamb well-being and guide mechanical ventilation. Scans were performed on a 3T MR scanner (Siemens Skyra, Erlangen, Germany) at Monash Biomedical Imaging, Monash University (Clayton, Australia), with a 15-channel radio frequency (RF) coil for both RF transmission and reception. The MRI protocol comprised structural imaging sequences (T1, T2, and DTI), susceptibility-weighted imaging (SWI), and a single-voxel MRS (6). The total acquisition time was about 40 min.

A 3D MPRAGE sequence was applied to achieve T1-weighted images, 102.5 mm thick, for whole brain coverage. The repetition time (TR) was 1,440 ms, with echo time (TE) of 3.92 ms, an inversion time of 900 ms, and a flip angle of 9°. The data matrix was 256 × 256 × 128, collected from a field of view (FOV) of 200 mm × 200 mm, giving a voxel size of 0.78 mm × 0.78 mm × 0.8 mm.

T2-weighted images with a 3D SPACE sequence, 44 mm thick, were also obtained to provide whole brain coverage (TR = 1,000 ms, TE = 132 ms, flip angle = 120°, and averages = 2). The matrix size was 384 × 384 × 88, collected from a FOV of 192 mm × 192 mm, giving a voxel size = 0.5 mm × 0.5 mm × 0.5 mm.

A standard 3D gradient echo sequence was used to acquire SWI for the whole brain with a slab thickness of 72 mm (TR = 28 ms, TE = 20 ms, and flip angle = 15°). The matrix size was 288 × 216 × 60, collected from a FOV of 176 mm × 132 mm, giving voxel size = 0.6 mm × 0.6 mm × 1.2 mm. The second phase encoding direction for 1.2-mm voxel was acquired in a parallel direction to the main magnetic field to optimise the susceptibility effect.

Diffusion tensor imaging with a spin-echo echo planar imaging sequence in the axial plane was obtained with 50 contiguous 1.2-mm slices (TR = 11,400 ms, TE = 99 ms). The data matrix was 128 × 128, collected from a FOV of 154 mm × 154 mm, giving a voxel size of 1.2 mm × 1.2 mm × 1.2 mm; for five *b*0. Diffusion encoding gradients were acquired in 30 directions with a *b* value of 1,000 s/mm^2^. The diffusion-weighted imaging was performed twice, and five *b* = 0 volumes were acquired.

A single-voxel spin-echo sequence was used to acquire MRS, localised on the supratentorial deep grey matter and central white matter (TR = 2,000 ms, TE = 270 ms) with a voxel size of 15 mm × 15 mm × 20 mm.

### MRI Data Analysis

MRI examination, image processing and analysis, and interpretation of the structural MR images were performed completely blind. Once ROI was drawn, the group allocation of each lamb was revealed for the final statistical analysis. The colour mapping approach was then completed, but not performed in a blinded fashion. Images acquired from T1, T2, and SWI scans were used to determine the presence of gross brain injury including infarcts and haemorrhages. DTI images were converted into NIFTI format, and then a brain mask was manually placed over the image using the FMRIB Software Library [FSL, FMRIB, Oxford, UK (27)]. DTI data were corrected for eddy-current distortion and then DTI parametric maps: fractional anisotropy (FA), axial diffusivity (AD), radial diffusivity (RD), and mean diffusivity (MD) were created with the Diffusion Toolbox of FMRIB. AD was used as the largest eigenvalue of the tensor, and RD was used as the average of the second and third eigenvalues of the tensor. All maps were then coregistered with high-resolution T2 images of the brains using Linear Image Registration Tool [FLIRT (28)]. The *b*0 image was used as the reference for the coregistration.

**Figure 1:**
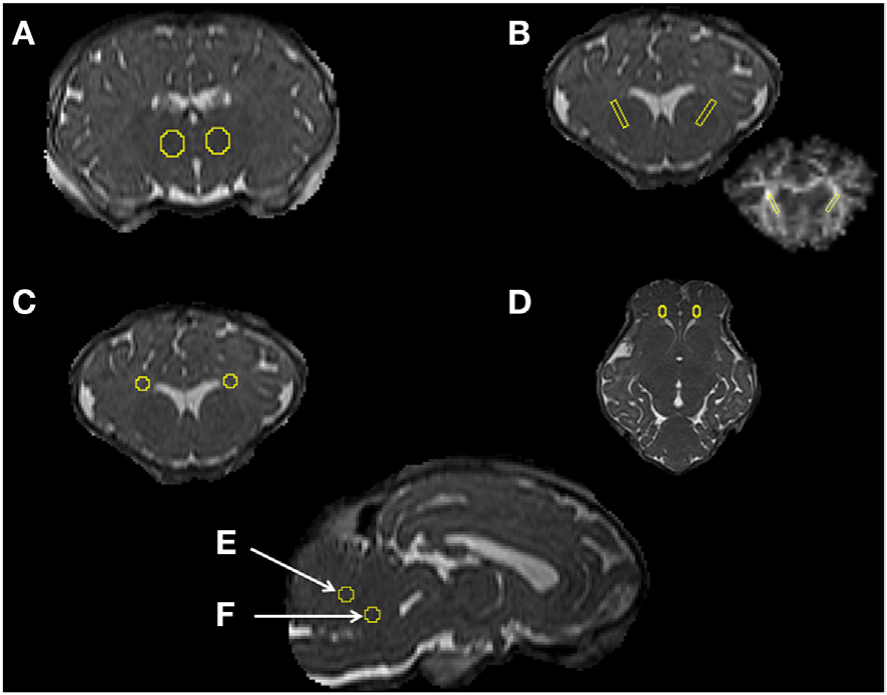
**Magnetic resonance imaging data analysis [regions of interest (ROIs)]**. Examples of ROIs in specific regions of the thalamus **(A)**, internal capsule **(B)**, periventricular white matter **(C)**, frontal white matter **(D)**, and the cerebellum vermis, targeting the midline and stalk white matter **(E,F)** for DTI analysis.

ROI were defined manually on high-resolution T2 images and then exported to the FA, AD, MD, and RD maps to calculate the region mean values for each lamb. ROIs were placed in specific regions including the thalamus (Th), periventricular white matter (PVWM), internal capsule (IC), frontal white matter (FWM), and the cerebellum (CB) vermis, targeting the stalk and midline white matter (Figure 1). The Sheep Brain Atlas from Michigan State University (Brain Biodiversity Bank, National Science Foundation) was used to facilitate accurate identification of the anatomical structures. The mean value of the adjacent four to five slices that defined a given ROI volume was calculated for each of the ROIs to test between-animal variability in diffusivity for both groups. MATLAB R2013a (MathWorks, MA, USA) was then used to calculate the SD for the ROIs of each region of the lamb brains to test within-animal variability (heterogeneity in DTI).

MRS data were processed using TARQUIN software to implement spectral fitting in the time domain (6). Peak area ratios for lactate (Lac) relative to *N*-acetylaspartate (NAA) (total of NAA and *N*-acetylaspartylglutamate), creatine (Cr; total of phosphocreatine and free creatine), and choline (Cho; total of glycerophosphocholine and phosphocholine) were calculated. In addition, we calculated peak area ratios for NAA/Cho, NAA/Cr, and Cho/Cr.

**Figure 2:**
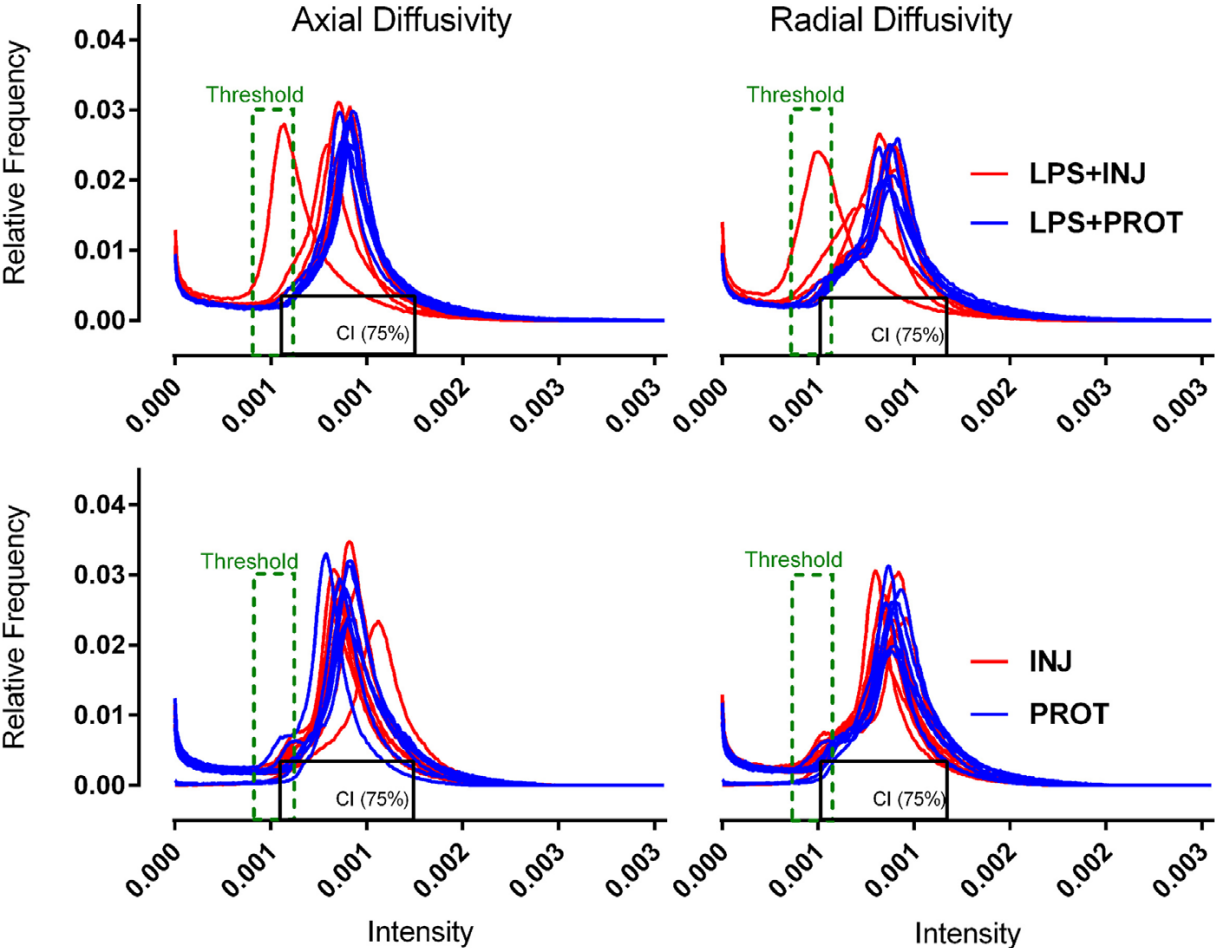
**Diffusion tensor imaging–histogram distributions**. Histogram distribution plots of axial diffusivity and radial diffusivity for the whole brains from each group. The red lines represent the injurious ventilation groups (LPS + INJ and INJ), while the blue lines represent the protective ventilation groups for both data sets (LPS + PROT and PROT). Black box represents the 75% CI of all voxel intensities observed in the distribution in protective lamb brains. Threshold (green dashed line) applied to the mapped intensities in the overlay in both groups. LPS, lipopolysaccharide; CI, confidence interval.

DTI data from the ROIs appeared to show more heterogeneity in the injured lamb brains. To further examine this variability, we utilised a colour map threshold technique to determine where the low diffusivity voxels were distributed in the DTI volumes. ImageJ (version 1.48, NIH, USA) was used to create a histogram of the distribution of the MR image pixel intensities at each position. For the purposes of comparison between animals, the histograms from all lambs in the two groups were normalised. The total pixel intensity distribution (histograms) for each lamb brain was compared between the minimum and maximum intensities within the brain for 256 bins in all DTI images. Histograms of the diffusivities for the whole brains of individual lambs in the two groups exposed to LPS were determined (Figure 2). Histograms were also plotted for the whole brains in the two groups of lambs that were not exposed to LPS in our previous study (6), as shown in (Figure 2). The histograms demonstrate the frequency distribution of MR image voxel intensity levels at each position. In the histograms, the black box represents 75% confidence interval (CI) of all voxel intensities observed in the distribution in protective lamb brains. The lower limit of this box was the lowest 10% of observed intensities. We then applied a threshold to the mapped intensities in the overlay in both groups (width ~10%). The upper threshold limit (Figure 2, green dashed line) was manually set at an intensity where voxels were almost completely absent across the whole brain in protective ventilation groups of lambs. However, coloured voxels were still evident at the edges of the brain mask in protective lambs. We found that the upper threshold of the colour mapping was consistent with the 90th percentile of intensities in the protective lambs, with 3–5% overlap in observed pixel intensities between protective and injurious ventilation groups (Figure 2). This process permitted mapping and quantification of the number of voxels of low diffusivity for AD, RD, and MD colour map images, using ImageJ.

### Statistical Analyses

Physiological data were compared using a two-way repeated measure ANOVA and Holm–Sidak *post hoc* analyses to determine significant interactions (Sigmaplot, Systat Software Inc.). The LPS + INJ group was compared to the LPS + PROT group to assess between-group differences in DTI and MRS parameters. The mean and SEM of voxel intensities within the predefined ROIs were calculated, and independent *t*-tests were used to compare treatment groups with the significance level set at *P* < 0.05. Data from the ROIs are presented as mean (box 5-95% CI of mean) with maximum − minimum error bars. Further, we calculated the SD for the ROIs in each lamb’s brain to test within-animal variability and to test the heterogeneity of DTI diffusivities for differences between groups using MATLAB (MathWorks Inc., MA, USA) software.

## Results

### Baseline Characteristics

LPS was successfully injected into the amniotic sac as confirmed by electrolyte analysis of amniotic fluid aspirated at the time of injection and the presence of thickened fetal membranes characteristic of this experimental intervention observed at delivery (12).

Mean gestational age (125 ± 2 days), body weights (LPS + PROT: 3.4 ± 0.6 kg; LPS + INJ: 3.6 ± 0.2 kg), and sex distributions of the lambs (LPS + PROT: 3 males and 3 females; LPS + INJ: 2 males and 4 females) were not different between groups.

### Physiological Parameters

**Figure 3:**
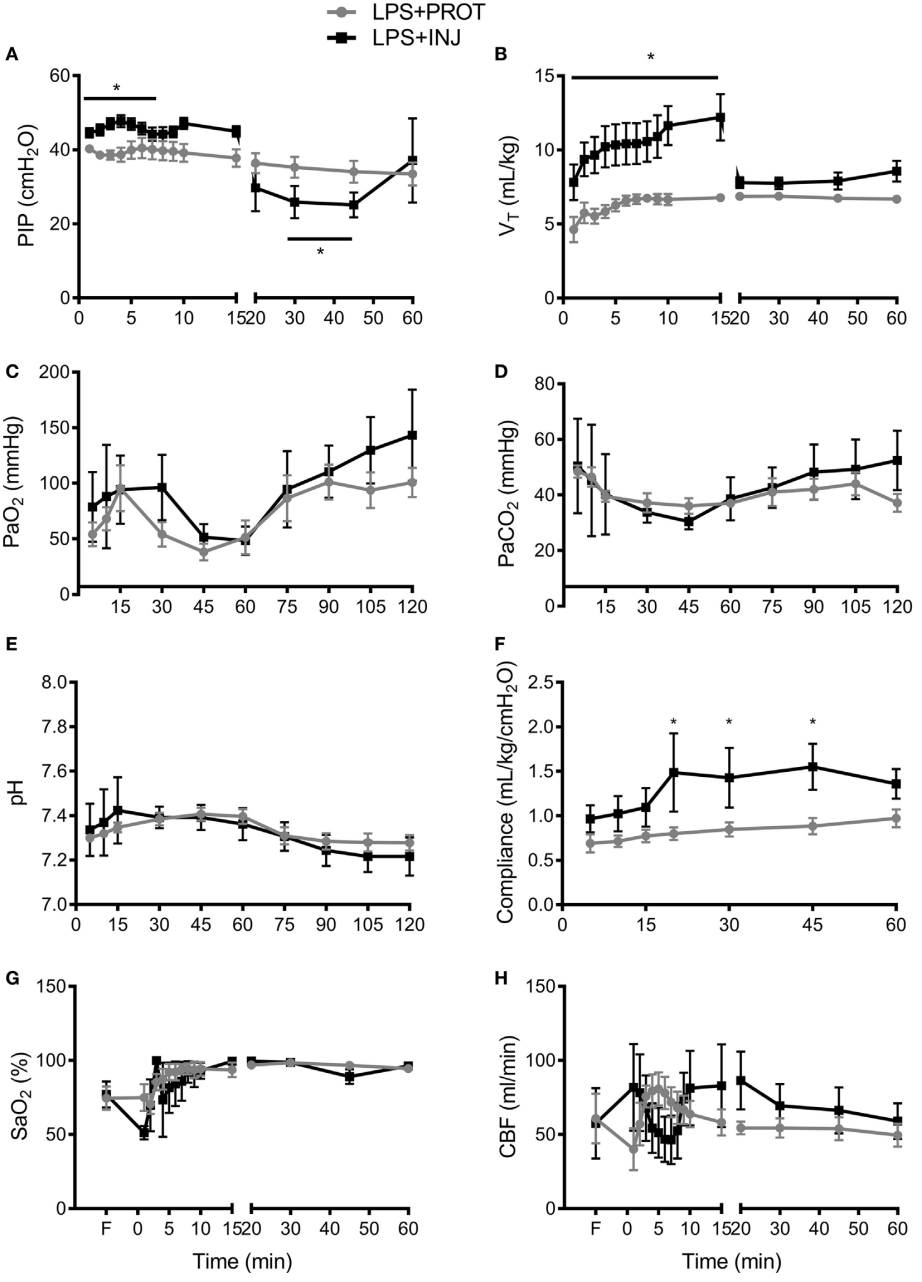
**Physiological parameters**. **(A)** Peak inspiration pressure (PIP) delivered to LPS + PROT lambs (closed circle) and LPS + INJ lambs (closed squares). **(B)** Tidal volume (V_T_). **(C)** Partial pressure of arterial oxygen (PaO_2_) and **(D)** carbon dioxide (PaCO_2_). **(E)** pH values. **(F)** Lung compliance. **(G)** Arterial oxygen saturation (SaO_2_). **(H)** Total carotid blood flow (CBF). *P < 0.01.

Physiological parameters were recorded for the initial 60 min, prior to transfer of the lamb into the MRI scanner. Peak inflation pressure (Figure 3A) was significantly higher in the LPS + INJ group than the LPS + PROT group during the first 15 min (*P* < 0.001), resulting in a significantly higher V_T_ during the initial 15 min than the LPS + PROT group (*P* < 0.001; Figure 3B). However, PIP was lower in LPS + INJ lambs once protective ventilation was initiated, likely due to the delivery of surfactant at 15 min in these lambs. Consistent with this contention, the LPS + INJ group demonstrated significantly increased lung compliance at 20, 30, and 45 min of ventilation (*P* = 0.02, *P* = 0.04, and *P* = 0.05, respectively; Figure 3F). PaO_2_, PaCO_2_, and pH were not different between groups (*P* = 0.38, *P* = 0.7, and *P* = 0.9, respectively; Figures 3C–E) nor was FiO_2_ (LPS + INJ; mean 0.41 ± 0.07 vs. LPS + PROT; mean 0.31 ± 0.06, *P* = 0.20). Oxygen saturation (SaO_2_) did not differ significantly between groups after the first 5 min of ventilation (*P* = 0.5; Figure 3G).

Carotid blood flow was variable within groups and between groups, but overall there was no significant differences observed (*P* = 0.8; Figure 3H). Mean HR tended to be lower at all time points in the LPS + INJ compared to LPS + PROT group (*P* = 0.09; data not shown).

### Structural MRI and MR Spectroscopy

**Figure 4:**
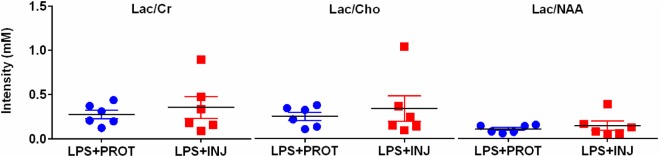
**Peak area MRS lactate ratios**. Individual MRS peak area ratios utilising a single-voxel encompassing supratentorial central white matter and deep grey matter in the LPS + PROT group (blue circles) and the LPS + INJ group (red squares). Lac, lactate; Cr, creatine; Cho, choline; NAA, *N*-acetylaspartate; MRS, magnetic resonance spectroscopy.

No overt cerebral injury was seen on T1, T2, or SWI images in either group. The MRS peak area ratios of Lac to other metabolites (Cr, Cho, and NAA) (Figure 4) as well as NAA/Cho, NAA/Cr, and Cho/Cr were not different between groups.

### DTI of ROI-Based Analysis

**Figure 5:**
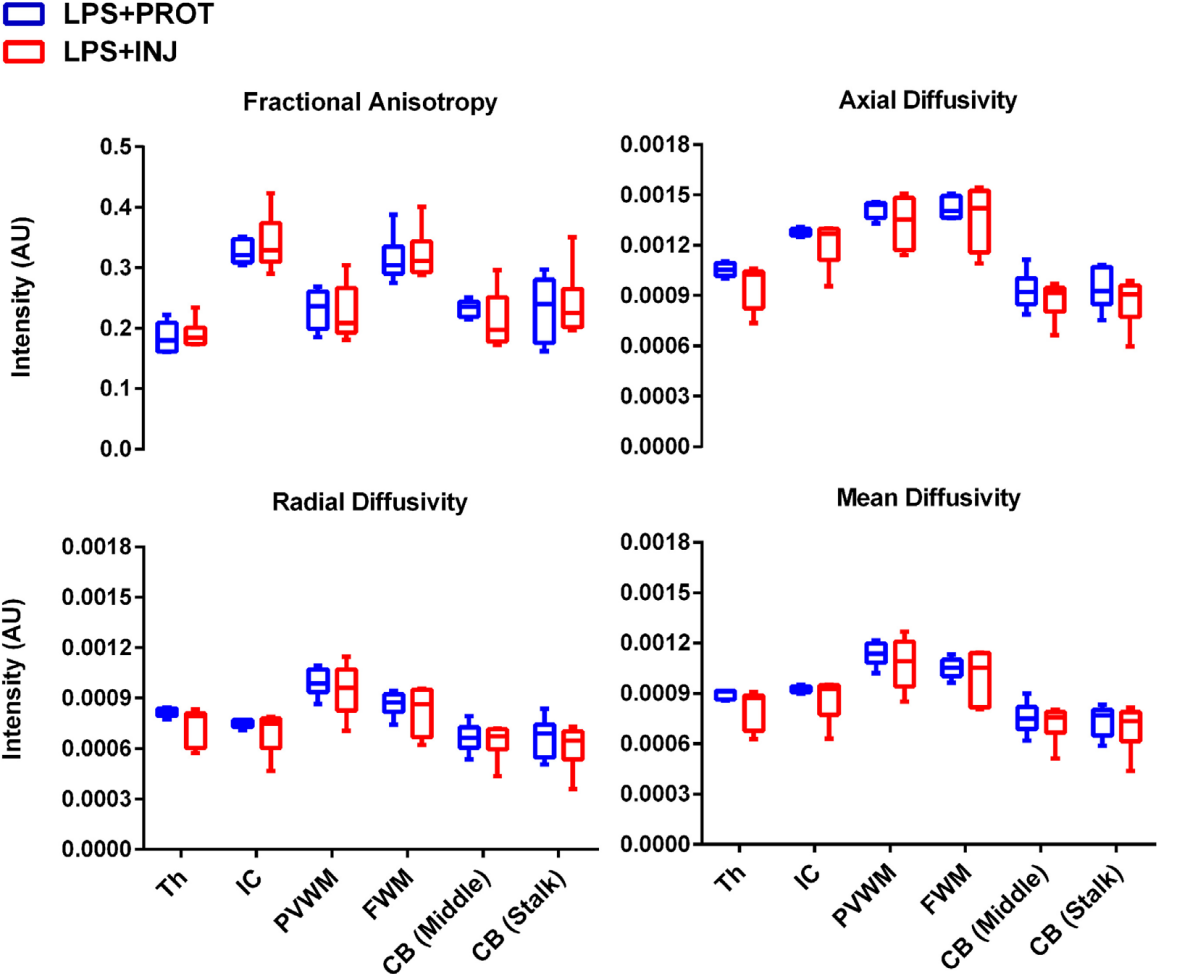
**Mean of diffusion tensor imaging measurements**. The mean fractional anisotropy, axial diffusivity, radial diffusivity, and mean diffusivity measurements for each region of interest (ROI) in the LPS + INJ and LPS + PROT groups. The ROIs shown in Figure 1 were located in the thalamus (Th), internal capsule (IC), periventricular white matter (PVWM), frontal white matter (FWM), cerebellum (CB) middle, and cerebellum (CB) stalk.

While mean AD, RD, and MD values were lower in the LPS + INJ lambs compared to the LPS + PROT lambs in many of the ROIs (Figure 5), no statistically significant differences were found. However, lambs in the LPS + INJ group showed significantly increased heterogeneity of diffusivity in multiple regions [Th, PVWM, and CB (middle)] compared to the LPS + PROT group (Figure S1 in Supplementary Material), indicating more animals with increased heterogeneity in their diffusivities within the ROIs. Given the greater variability in injury in the LPS + INJ group, we applied a colour map threshold technique to better distinguish between the two ventilation groups.

### Colour Mapping Threshold of Whole Brain Analysis

**Figure 6:**
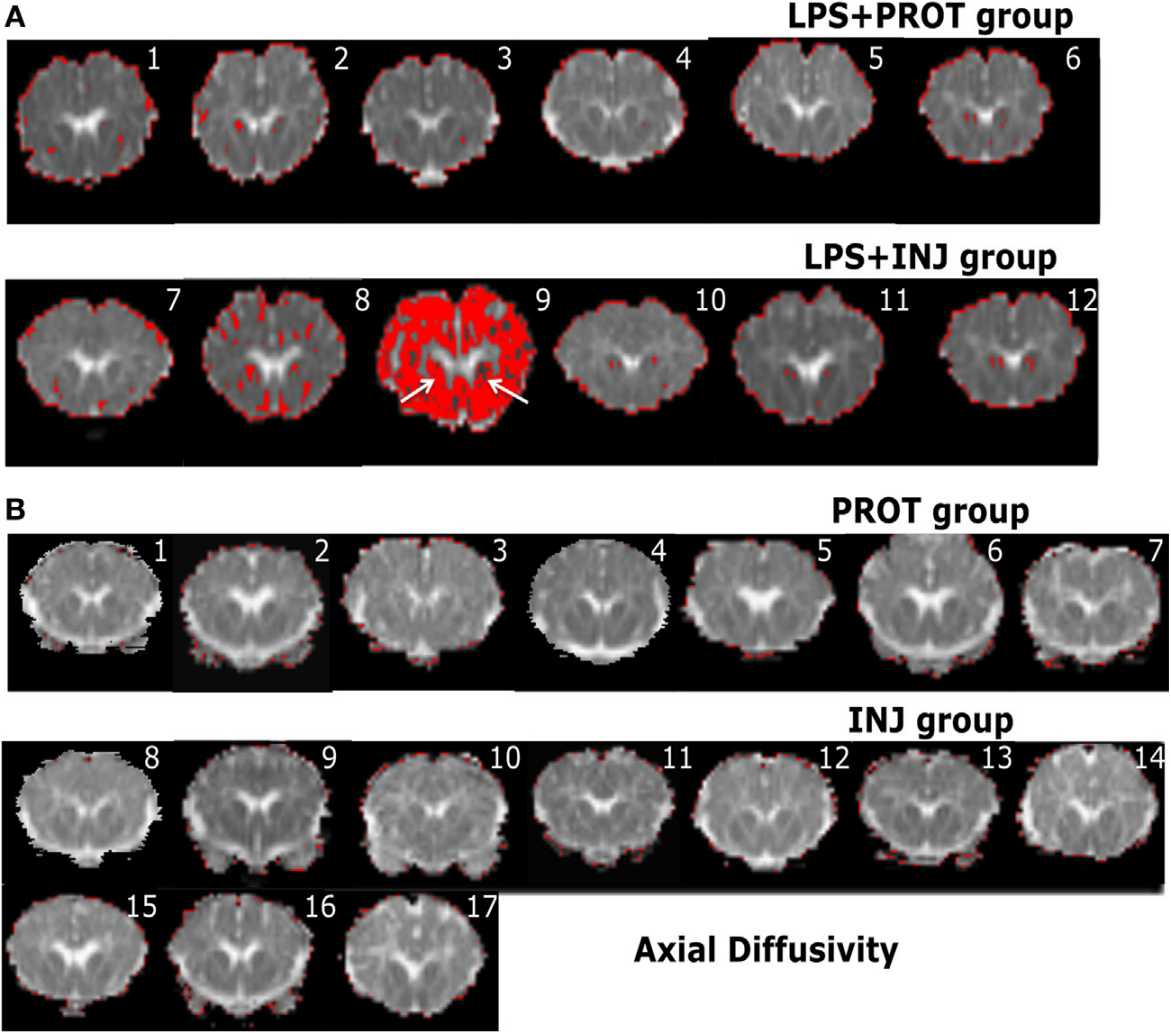
**Example whole brain diffusion tensor imaging-colour maps of the internal capsule (IC) and axial diffusivity (AD)**. Voxel diffusivity intensities falling below the low threshold are shown as red for AD measurements for all lambs exposed to intrauterine inflammation [lipopolysaccharide (LPS) **(A)**] and control lambs not exposed to LPS **(B)**. In the LPS + INJ, all low-diffusion maps are overlaid on diffusion images for a slice passing through the IC. While the red colour indicates lower values in the range of the threshold, the black colour indicates lower values below the threshold shown in Figure 2. In the control groups, INJ and PROT, no lamb had low AD values in the IC, putamen, caudate, and septum in either group. Numerals in images indicate the ID for individual lambs.

**Figure 7:**
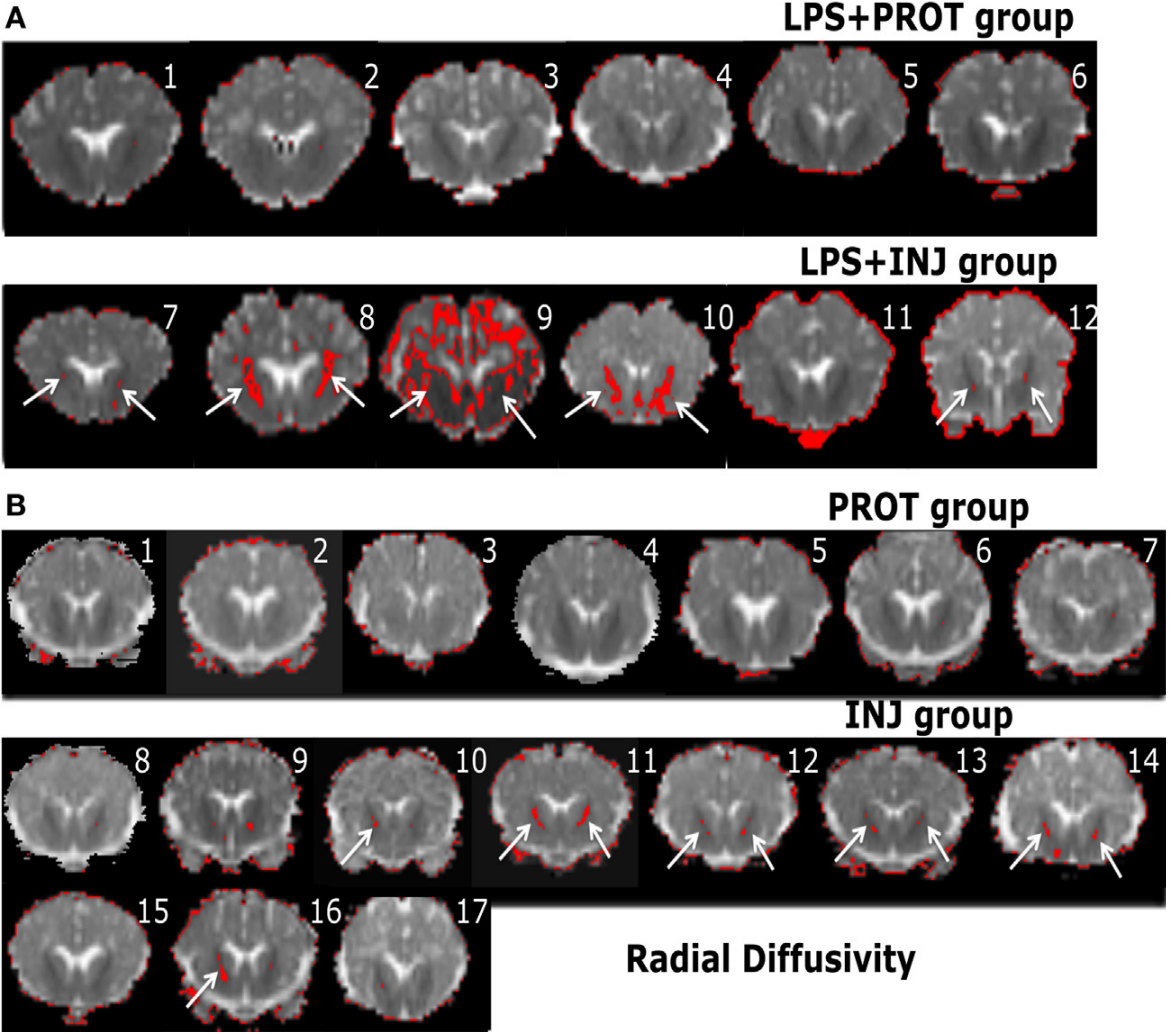
**Example whole brain diffusion tensor imaging-colour maps of the internal capsule (IC) and radial diffusivity (RD)**. Voxel diffusivity intensities falling below the low threshold are shown as red for RD measurements for all lambs exposed to intrauterine inflammation [lipopolysaccharide (LPS) **(A)**] and control lambs not exposed to LPS **(B)**. In the LPS + INJ, all low-diffusion maps are overlaid on diffusion images for a slice passing through the IC. While the red colour (IC indicated by white arrows) indicates lower values in the range of the threshold, the black colour indicates lower values below the threshold shown in Figure 2. In the control groups, INJ and PROT, the colour images display low diffusivity in the IC for some INJ lambs, while there were no low RD values found in the PROT group. Numerals in images indicate the ID for individual lambs.

Colour map techniques were used to describe the tensor orientation in relation to the white matter tract direction (29). Histograms of AD and RD for the whole brains of individual lambs in the two groups exposed to LPS and two groups without LPS treatment (6) are plotted in Figure 2. The threshold range that highlighted lower diffusivity voxels (Figure 2, green dashed line) was manually set below the 90th percentile of intensities in the protective lambs and hence corresponded to the red shading in the injured lambs as shown in the examples of Figures 6 and 7.

In the low diffusivity maps obtained from the lambs exposed to LPS during the perinatal period, the red shading (threshold range) was more visible in the Th of AD and RD maps (Figure S2 in Supplementary Material) in three LPS + INJ lambs, while the lowest diffusivity values were almost absent in the same region in the LPS + PROT group. Further, there were low AD and RD values in the amygdala of one LPS + INJ lamb (Figure S2 in Supplementary Material). In the FWM, there were three LPS + INJ lamb brains that showed lower AD values compared to those in the LPS + PROT group (Figure S3 in Supplementary Material). Further, low RD values appeared in the FWM of two LPS + INJ lambs compared to the LPS + PROT group lambs (Figure S3 in Supplementary Material). We also found exceptionally low AD and RD voxel values, as shown in red and black (below the threshold range), in the IC, putamen, caudate, and septum of lambs in the LPS + INJ group, but rarely in the LPS + PROT group (Figures 6 and 7). In the PVWM, low AD and RD voxel diffusivities were found in the LPS + INJ group when compared to LPS + PROT group lambs in addition to grey matter regions of the cerebellum, which were lower in diffusivity than white matter (Figure S4 in Supplementary Material). In all ROIs, the calculated MD maps had similar patterns of distribution of low diffusivity as shown in the AD and RD maps (Figures S4 and S5 in Supplementary Material). Therefore, the colour maps revealed that the low-intensity voxels for AD, RD, and MD closely correlated with white matter regions in most of the cerebral anatomical structures that were chosen.

### Colour Mapping Threshold Comparison with Control Lambs

**Figure 8:**
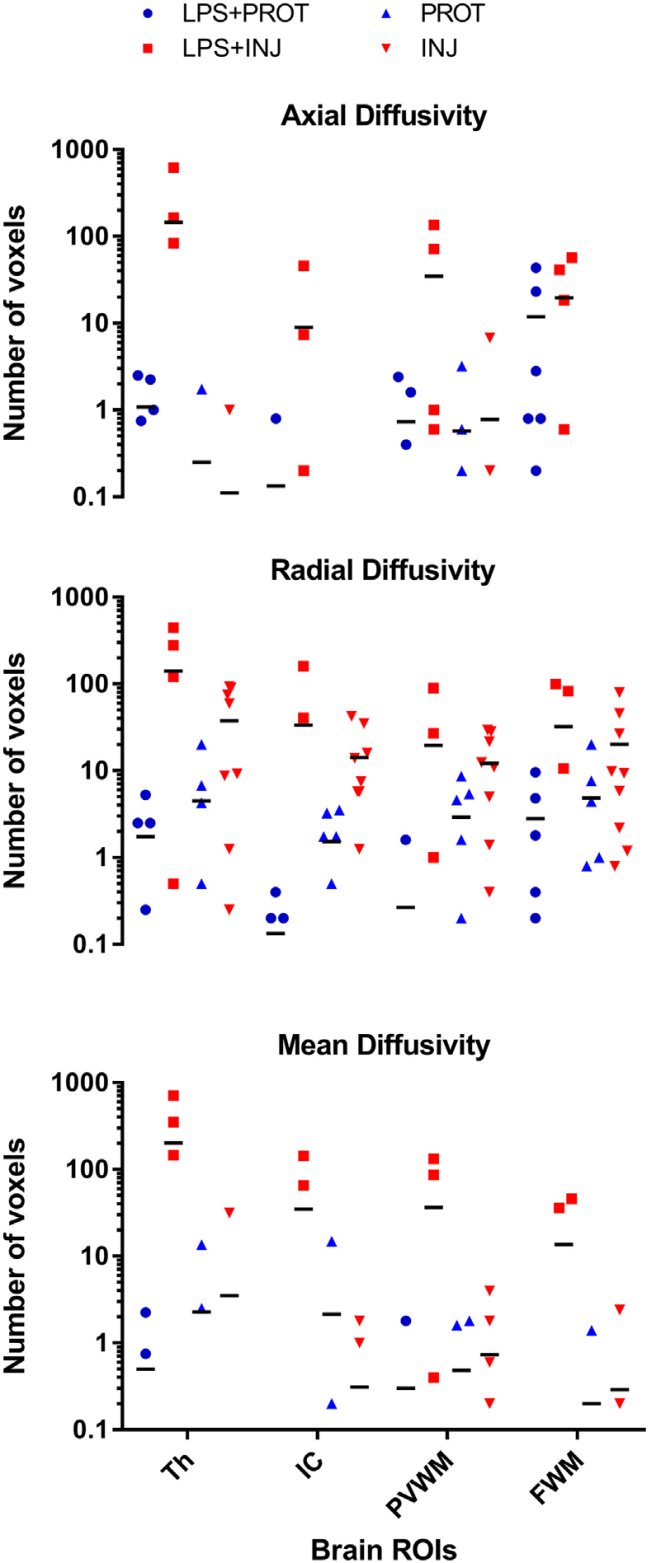
**Comparison of voxel-based colour map image**. The comparison of voxel-based colour images for axial diffusivity (AD), radial diffusivity (RD), and mean diffusivity (MD) measurements for each region of interest (ROI) in the lamb brains for both data sets. The ROIs shown in Figure 1 were located in the thalamus (Th), internal capsule (IC), periventricular white matter (PVWM), and frontal white matter (FWM). There were widespread low RD intensity voxels in all white matter regions in both groups of lambs exposed to intrauterine inflammation [lipopolysaccharide (LPS)], but low AD and MD intensity voxels were more frequent in the LPS + INJ group only. In contrast, lambs that were not exposed to intrauterine inflammation (LPS) were found to have few low RD intensity voxels in white matter regions, and no change was found in the AD and MD intensities.

As a control reference, we applied the colour map technique to lambs that underwent the same ventilator and MRI protocols but were not exposed to LPS (PROT and INJ control lambs); the physiology and other MRI data have been reported previously (6). Lambs from our previous study did not differ in body weight, blood gas parameters, or HR at the time of intubation and cord clamping (time 0; Table 1) with the lambs from this study. We found no low AD values in the FWM, IC, putamen, caudate, septum, and PVWM for either PROT or INJ groups, while low RD values appeared in some regions of white matter of INJ lambs when compared to PROT group (Figures S6 and S7 in Supplementary Material). Finally, quantifying the number of voxels found to have low diffusivity within each ROI, we compared counts between LPS and control lambs (Figure 8). There were frequently lower AD, RD, and MD intensities in the Th, IC, PVWM, and FWM in a subgroup of LPS + INJ lambs compared to LPS + PROT, as well as when compared to controls when compared to controls that were not exposed to LPS.

**Table 1:** **Comparison of blood gas parameters at the time of delivery between the lambs that were exposed to intrauterine inflammation (LPS) and controls lambs that were not exposed to LPS at the delivery time**.

Variable	PROT	INJ	LPS + PROT	LPS + INJ	*P* value
Sex (M/F)	4/3	4/6	3/3	2/4	–
Gestational age (days)	126 ± 1	126 ± 1	125 ± 2	125 ± 2	0.9
Body weight (kg)	3.3 ± 0.2	3.2 ± 0.2	3.4 ± 0.4	3.6 ± 0.3	0.09
SpO_2_ (%)	71.7 ± 4.2	79.3 ± 11.6	75.3 ± 22.8	61 ± 26.9	0.5
Heart rate (bpm)	158.7 ± 24.5	130.0 ± 3.5	128.7 ± 10.7	145.8 ± 33.8	0.3
pH	7.3 ± 0.7	7.2 ± 0.7	7.3 ± 0.5	7.2 ± 0.6	0.2
PaCO_2_ (mmHg)	51.4 ± 11.1	64.5 ± 0.8	57.2 ± 15.5	54.2 ± 15.4	0.4
PaO_2_ (mmHg)	62.4 ± 17.7	23.5 ± 0.8	42 ± 28.9	48.2 ± 54.3	0.2

*Characteristics of the PROT and INJ lambs from the study by Skiold et al. (6) and LPS + PROT and LPS + INJ lambs in this study and their fetal blood gases at the time of intubation and cord clamping are presented as mean ± SD. Variables were compared using one-way ANOVA with a Holm-Sidak’s multiple comparisons tests relative to the PROT group. *P* < 0.05 was considered statistically significant*.

*PROT, protective ventilation; INJ, injurious ventilation; LPS, lipopolysaccharide*.

## Discussion

Preterm neonates exposed to intrauterine inflammation are at an increased risk of ventilation-induced brain inflammation and injury (11). In this study, we aimed to detect subtle brain injury in lambs associated with intrauterine inflammation and different ventilation strategies within the first 90 min after birth, using clinical 3T MRI. We found that conventional MRI techniques were unable to detect subtle differences in brain injury between LPS groups. However, colour mapping demonstrated that there were more regions of low diffusivity in a subgroup of LPS + INJ lambs compared to LPS + PROT lambs, indicative of increased brain injury. Further, exposure to LPS resulted in consistently increased regions of low diffusivity than control lambs, suggesting that early detection of brain injury in infants exposed to chorioamnionitis may be possible.

Advanced MRI methods such as DTI and MRS provide useful information about structural connectivity and altered brain chemistry, allowing improved detection of newborn brain injury over conventional MRI (30–33). However, these strategies have limited ability to detect brain injury early enough whereupon it can guide clinical intervention within a therapeutic window (24, 25). This contention is confirmed by our finding that conventional MRI sequences revealed no evidence of brain injury in the preterm lambs that had received LPS with or without injurious ventilation, despite histological studies demonstrating that injury is normally present (11). Therefore, there is an urgent need to develop advanced MRI strategies that can detect subtle brain injury early after birth. Further, clinicians need to be confident that brain injury is developing, or already present, prior to administration of any therapy.

Several other research groups have presented evidence of DTI changes due to brain injury in lambs utilising *ex vivo* MRI analyses that were strongly correlated with histopathological evidence of injury (34–36). The colour map threshold approach was used in this *in vivo* study to measure the distribution of diffusivity values in specific brain regions that have been identified as being vulnerable to injury. The advantage of colour mapping is that it enables the analysis of thousands of voxels compared with very small numbers of voxels when using small defined ROIs (37). We used lambs from a previous study, which received protective ventilation after preterm delivery to determine the ~10% threshold, as these lambs were most likely to have very little brain injury (6). By using this new technique, we found that lower AD, RD, and MD intensities are more frequently seen in the Th, FWM, IC, and PVWM of LPS + INJ lambs compared to LPS + PROT lambs. We then compared these data with that of lambs from our previous study (6), which were not exposed to LPS. We observed that lambs exposed to chronic intrauterine inflammation more frequently had low diffusivity in the Th, FWM, IC, and PVWM regions when compared with lambs not exposed to intrauterine inflammation. Therefore, threshold mapping of DTI is a more sensitive approach to assess the microstructural changes associated with preterm brain injury as early as 90 min after birth.

The colour map technique identified decreased AD, RD, and MD in LPS + INJ lambs, suggestive of microstructural alterations in many ROIs. The decrease in AD indicates a decrease in water diffusion parallel to white matter fibre tracts, suggesting that there is a breakdown of axon integrity within these ROIs (38). The reduction in RD, reflecting the degree of water diffusion perpendicular to white matter fibre tracts in anatomical brain structures, also suggests that myelin might be affected (38). This observation is consistent with a previous observation that LPS exposure decreased myelination in fetal sheep (39). Nonetheless, a limitation of the colour mapping approach is that it was not sensitive enough to detect significant differences between the groups in the cerebellum. This may be attributed to fibre tracts being less organised and only partially myelinated (40). For this reason, the cerebellum may be less sensitive to the effects of inflammation. Further, the water content in the premature brain is more uniform than later on in development (41), diminishing the MR contrast between the white matter and grey matter in some regions, such as the cerebellum (42).

In this study of chronic intrauterine inflammation, a subgroup of LPS + INJ lambs had brain injury of greater extent than the LPS + PROT group. In contrast, our previous study utilising a protective ventilation strategy did not find a reduction in the degree or severity of white matter injury in preterm lambs exposed to 2 days of LPS (12). It is most likely that this disparity reflects differences in the methods of assessment of brain injury utilised in the two studies. However, other studies suggest that part of the difference between the past study (12) and this study is likely to be also due to the longer time (7 days) of LPS exposure, which may increase the susceptibility of the brain to ventilation-induced injury. Previously we found increased incidence and severity of white matter injury in ventilated preterm lambs receiving LPS 4 days prior to delivery compared to 2 days (11). Importantly, our evidence that changes in brain diffusion was greater in LPS-exposed lambs than controls supports both our findings (5) and clinical studies (13) demonstrating an increased risk of postnatal brain injury after intrauterine inflammation.

We did not find differences in MRS peak area ratios for Lac relative to other metabolites (Cr, Cho, and NAA) between PROT and INJ groups following LPS administration. These ratios are indicative of brain metabolism and correlate with neonatal brain injury (43) and subsequent outcome (44). We do not know why we were unable to detect differences using MRS, but the prior exposure to LPS may alter the response of the metabolites to ventilation.

There are some limitations to this study. We did not correlate our quantitative DTI findings with histological assessment to conclusively demonstrate that these alterations truly represent microstructural changes exacerbated by multiple risk factors such as injurious ventilation and chronic inflammation. Further, conducting longer term studies is required to investigate whether the early injury markers manifest as disability later in life. Moreover, we concede that the greatest limitation of the application of this imaging and analysis approach to very preterm and very low-birth-weight neonates in the NICU is that it is difficult to image ventilated babies in an MR scanner within hours of birth, and this would be discouraged by the general policy of minimal handling of such neonates. However, if we can develop robust strategies that can accurately determine brain injury, then the potential benefit of early identification and treatment would likely outweigh the minimal handling policies of such units. The use of new MRI-compatible cribs might make such early diagnosis easier to implement.

In conclusion, the use of colour mapping demonstrated that injurious ventilation after LPS was associated with low AD, RD, and MD in the cerebral white matter of preterm lambs, which was not detected using standard MRI techniques. Colour mapping also demonstrated greater alterations in water diffusivity, and therefore neural function, in LPS lambs compared to controls. The colour map technique might be a sensitive way to detect early white matter injury and may lead to improved detection of preterm brain injury within a suitable time frame for clinical intervention.

## Author Contributions

All authors made substantial contributions to the conception or design of the work, or the acquisition, analysis, or interpretation of data for the work; revised drafts; and approved the final version to be published.

## Conflict of Interest Statement

The funders had no role in study design, data collection and analysis, decision to publish, or preparation of the manuscript. The authors declare that they do not have any conflicts of interests.

## Supplementary Material

Figure S1 **SD of diffusion tensor imaging measurements**. The SD of the distribution of voxel intensities within each region of interest (ROI) for each animal was calculated for fractional anisotropy, axial diffusivity, radial diffusivity, and mean diffusivity measurements in the LPS + INJ and LPS + PROT groups. The means of the SDs were tested for differences between the two groups of lambs. The ROI (see Figure 1) were located in the thalamus (Th), internal capsule (IC), periventricular white matter (PVWM), frontal white matter (FWM), cerebellum (CB) middle, and cerebellum (CB) stalk. **P* < 0.01 and ***P* < 0.001.

Figure S2 **Diffusion tensor imaging-colour maps of the thalamus; lipopolysaccharide (LPS) groups**. Voxel diffusivity intensities falling below the low threshold are shown as red for axial diffusivity (AD) and radial diffusivity (RD) measurements for all lambs exposed to intrauterine inflammation (LPS). All low-diffusion maps are overlaid on diffusion images for a slice passing through the thalamus. The red overlay (threshold range) was more visible in the thalamus of AD and RD maps in three LPS + INJ lambs (#7–9), while the lowest diffusivity values were almost absent in the same region in the LPS + PROT group. Further, there were low AD and RD values in the amygdala of one LPS + INJ lamb (#9). Numerals in images indicate the ID for individual lambs.

Figure S3 **Diffusion tensor imaging-colour maps of the frontal white matter (FWM); lipopolysaccharide (LPS) groups**. Voxel diffusivity intensities falling below the low threshold are shown as red for axial diffusivity (AD) and radial diffusivity (RD) measurements for all lambs exposed to intrauterine inflammation (LPS). All low-diffusion maps are overlaid on diffusion images for a slice passing through the FWM. While the red colour indicates lower values in the range of the threshold (arrows indicating FWM), the black colour indicates voxel intensity below the threshold range shown in Figure 2. There were three LPS + INJ lamb brains (#7–9) that showed lower AD values in the FWM compared to those in the LPS + PROT group. Further, low RD values appeared in the FWM of two LPS + INJ lambs (#8 and 9) compared to the LPS + PROT group lambs. Numerals in images indicate the ID for individual lambs.

Figure S4 **Diffusion tensor imaging-colour maps of the periventricular white matter (PVWM); lipopolysaccharide (LPS) groups**. Voxel diffusivity intensities falling below the low threshold are shown as red for axial diffusivity, radial diffusivity, and mean diffusivity measurements for all lambs exposed to intrauterine inflammation (LPS). All low-diffusion maps are overlaid on diffusion images for a slice passing through the PVWM. The red shows the lower diffusivities values in the range of the threshold, while the black colour indicates diffusivities values below the threshold (Figure 2). While noting the red colour in the grey matter regions, which have lower diffusivity than white matter, there are two. LPS + INJ lambs (#8 and 9) with widespread lower diffusivity values in the PVWM when compared to LPS + PROT group lambs. Numerals in images indicate the ID for individual lambs.

Figure S5 **Diffusion tensor imaging-colour maps of the mean diffusivity (MD) in the thalamus; lipopolysaccharide (LPS) groups**. Voxel diffusivity intensities falling below the low threshold are shown as red for MD, measurements for all lambs exposed to intrauterine inflammation (LPS). All low-diffusion maps are overlaid on diffusion images for a slice passing through the thalamus. The red colour in the images shows low MD that is more visible in the thalamus of three injured lambs (LPS + INJ) (#7–9), while the MD images of the LPS + PROT group show very few red voxels. There were reduced MD values in amygdala of one injured lamb as well (#9). Numerals in images indicate the ID for individual lambs.

Figure S6 **Diffusion tensor imaging-colour maps of the frontal white matter (FWM); control groups**. Voxel diffusivity intensities falling below the low threshold are shown as red for axial diffusivity (AD) and radial diffusivity (RD) measurements for all control lambs not exposed to intrauterine inflammation (lipopolysaccharide). All low-diffusion maps are overlaid on diffusion images for a slice passing through the FWM. None of the lambs in either group had low AD values in the FWM, but the red colour is evident in the grey matter in some animal of both groups. In contrast, low RD values appeared in the FWM of three INJ lambs (#9, 11, and 12). Numerals in images indicate the ID for individual lambs.

Figure S7 **Diffusion tensor imaging-colour maps of the periventricular white matter (PVWM); control groups**. Voxel diffusivity intensities falling below the low threshold are shown as red for axial diffusivity (AD) and radial diffusivity (RD) measurements for all control lambs not exposed to intrauterine inflammation (lipopolysaccharide). All low-diffusion maps are overlaid on diffusion images for a slice passing through the PVWM. There were no low AD diffusivity voxels concentrated in the PVWM in either group of lambs, but there were four INJ lambs (#9, 11, 13, and 16) with widespread low RD voxels in the PVWM when compared to PROT group lambs. Numerals in images indicate the ID for individual lambs.

## Abbreviations

AD, axial diffusivity; DTI, diffusion tensor imaging; FA, fractional anisotropy; IA, intra-amniotic; INJ, injurious ventilation; LPS, lipopolysaccharide; MD, mean diffusivity; MRI, magnetic resonance imaging; MRS, magnetic resonance spectroscopy; NAA, *N*-acetylaspartate; PROT, protective ventilation; RD, radial diffusivity; RF, radio frequency; SWI, susceptibility-weighted imaging; T_1_, spin–lattice relaxation time; T_2_, spin–spin relaxation time.
