# Supplementary figures and images for: Diffusion Tensor Imaging Colour Mapping Threshold for Identification of Ventilation-Induced Brain Injury after Intrauterine Inflammation in Preterm Lambs
Source: Front Pediatr. 2017 Apr 5;5:70. doi: 10.3389/fped.2017.00070 (PMC5380678; doi:10.3389/fped.2017.00070)

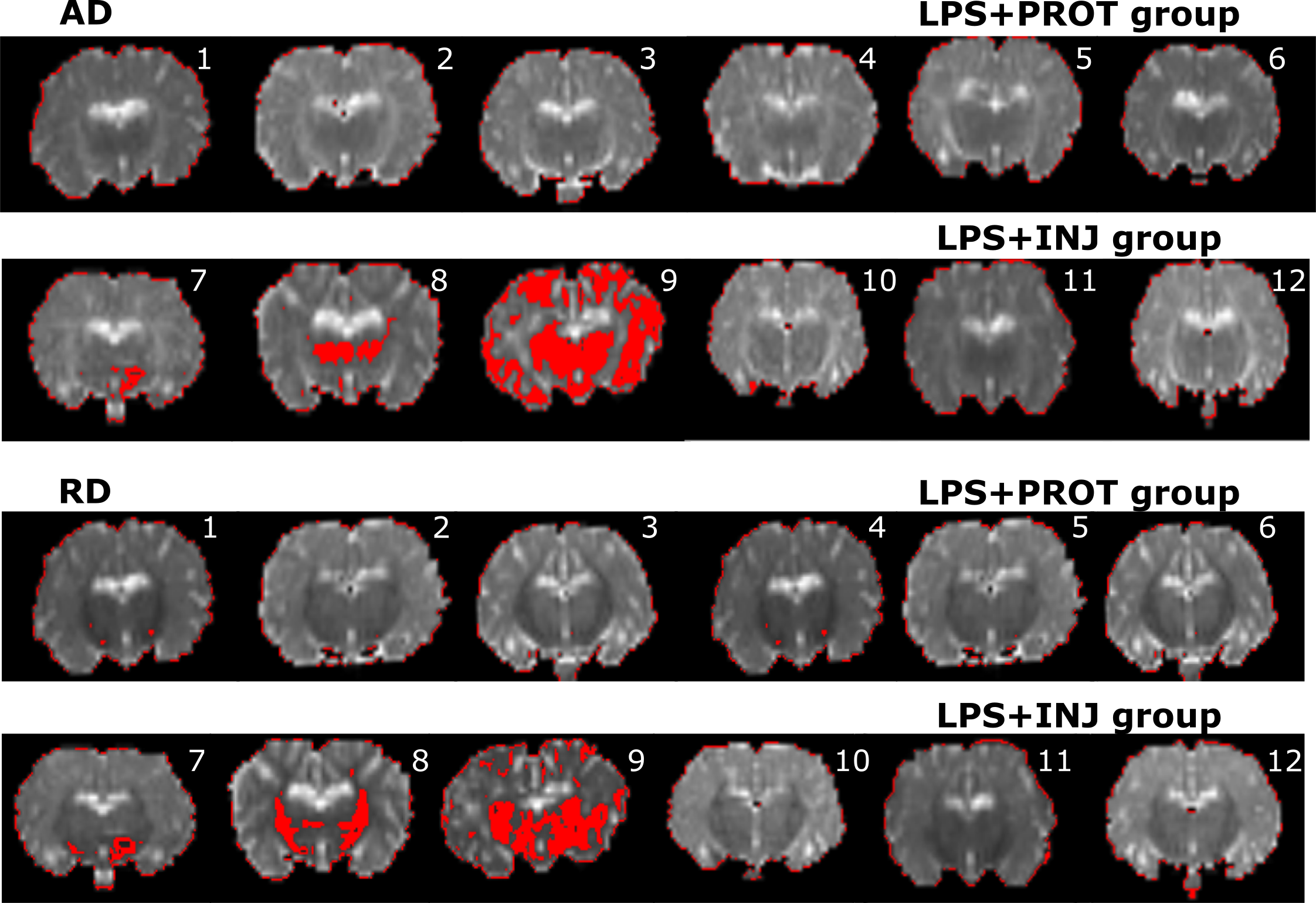

Supplement: Figure S2 — Diffusion tensor imaging-colour maps of the thalamus; lipopolysaccharide (LPS) groups. Voxel diffusivity intensities falling below the low threshold are shown as red for axial diffusivity (AD) and radial diffusivity (RD) measurements for all lambs exposed to intrauterine inflammation (LPS). All low-diffusion maps are overlaid on diffusion images for a slice passing through the thalamus. The red overlay (threshold range) was more visible in the thalamus of AD and RD maps in three LPS + INJ lambs (#7–9), while the lowest diffusivity values were almost absent in the same region in the LPS + PROT group. Further, there were low AD and RD values in the amygdala of one LPS + INJ lamb (#9). Numerals in images indicate the ID for individual lambs. [file Image_2.TIF]

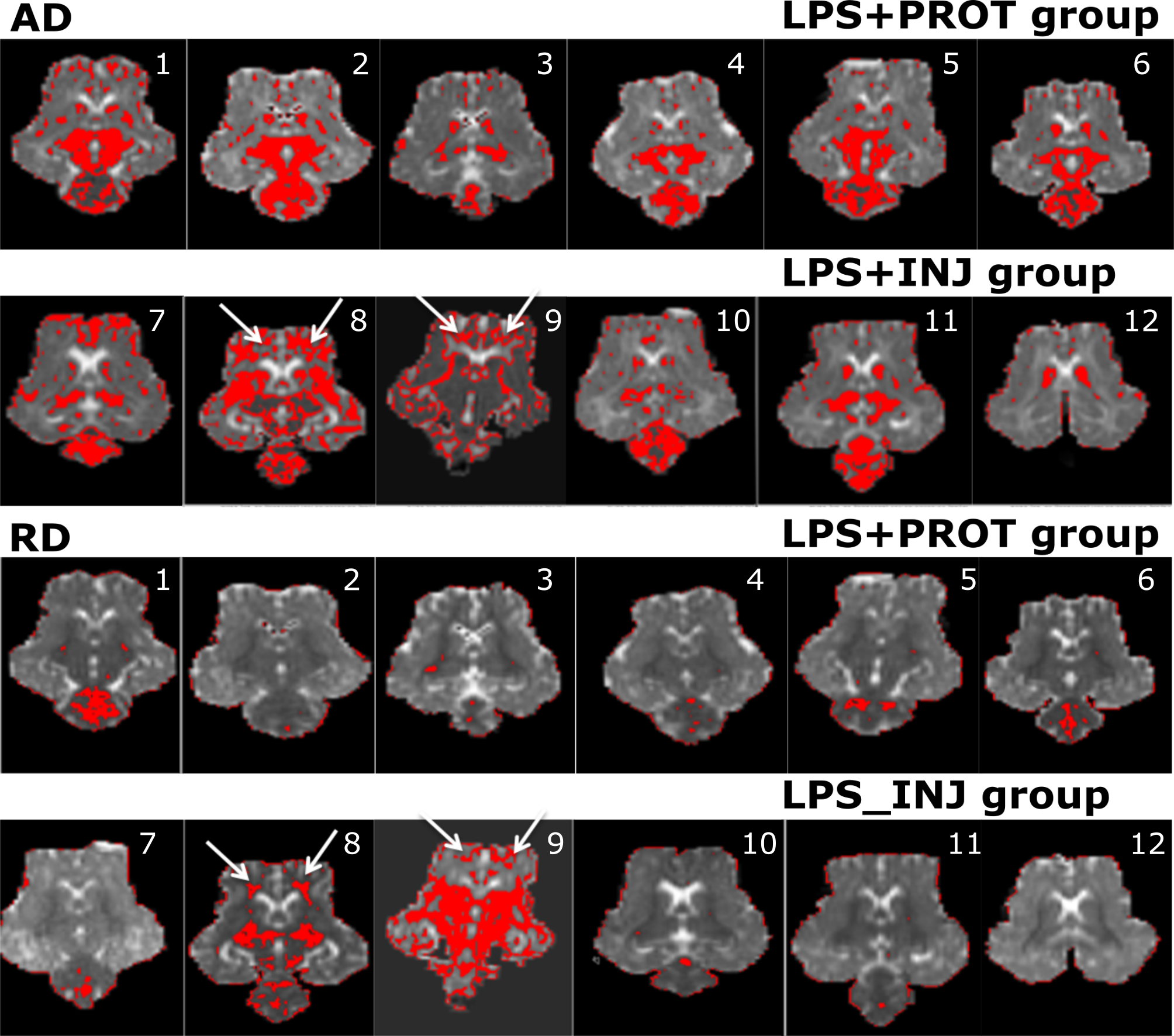

Supplement: Figure S3 — Diffusion tensor imaging-colour maps of the frontal white matter (FWM); lipopolysaccharide (LPS) groups. Voxel diffusivity intensities falling below the low threshold are shown as red for axial diffusivity (AD) and radial diffusivity (RD) measurements for all lambs exposed to intrauterine inflammation (LPS). All low-diffusion maps are overlaid on diffusion images for a slice passing through the FWM. While the red colour indicates lower values in the range of the threshold (arrows indicating FWM), the black colour indicates voxel intensity below the threshold range shown in Figure 2. There were three LPS + INJ lamb brains (#7–9) that showed lower AD values in the FWM compared to those in the LPS + PROT group. Further, low RD values appeared in the FWM of two LPS + INJ lambs (#8 and 9) compared to the LPS + PROT group lambs. Numerals in images indicate the ID for individual lambs. [file Image_3.TIF]

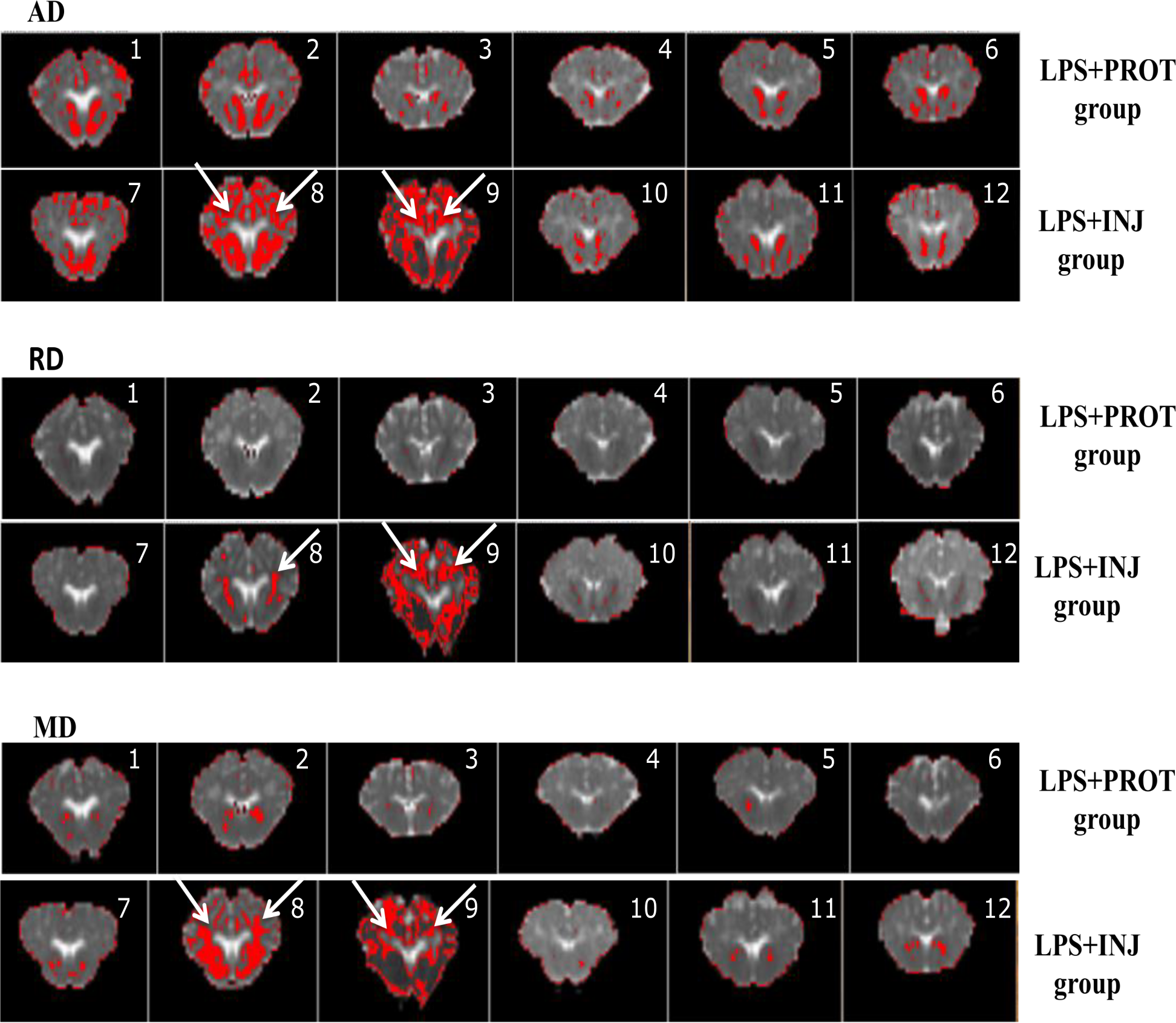

Supplement: Figure S4 — Diffusion tensor imaging-colour maps of the periventricular white matter (PVWM); lipopolysaccharide (LPS) groups. Voxel diffusivity intensities falling below the low threshold are shown as red for axial diffusivity, radial diffusivity, and mean diffusivity measurements for all lambs exposed to intrauterine inflammation (LPS). All low-diffusion maps are overlaid on diffusion images for a slice passing through the PVWM. The red shows the lower diffusivities values in the range of the threshold, while the black colour indicates diffusivities values below the threshold (Figure 2). While noting the red colour in the grey matter regions, which have lower diffusivity than white matter, there are two. LPS + INJ lambs (#8 and 9) with widespread lower diffusivity values in the PVWM when compared to LPS + PROT group lambs. Numerals in images indicate the ID for individual lambs. [file Image_4.TIF]

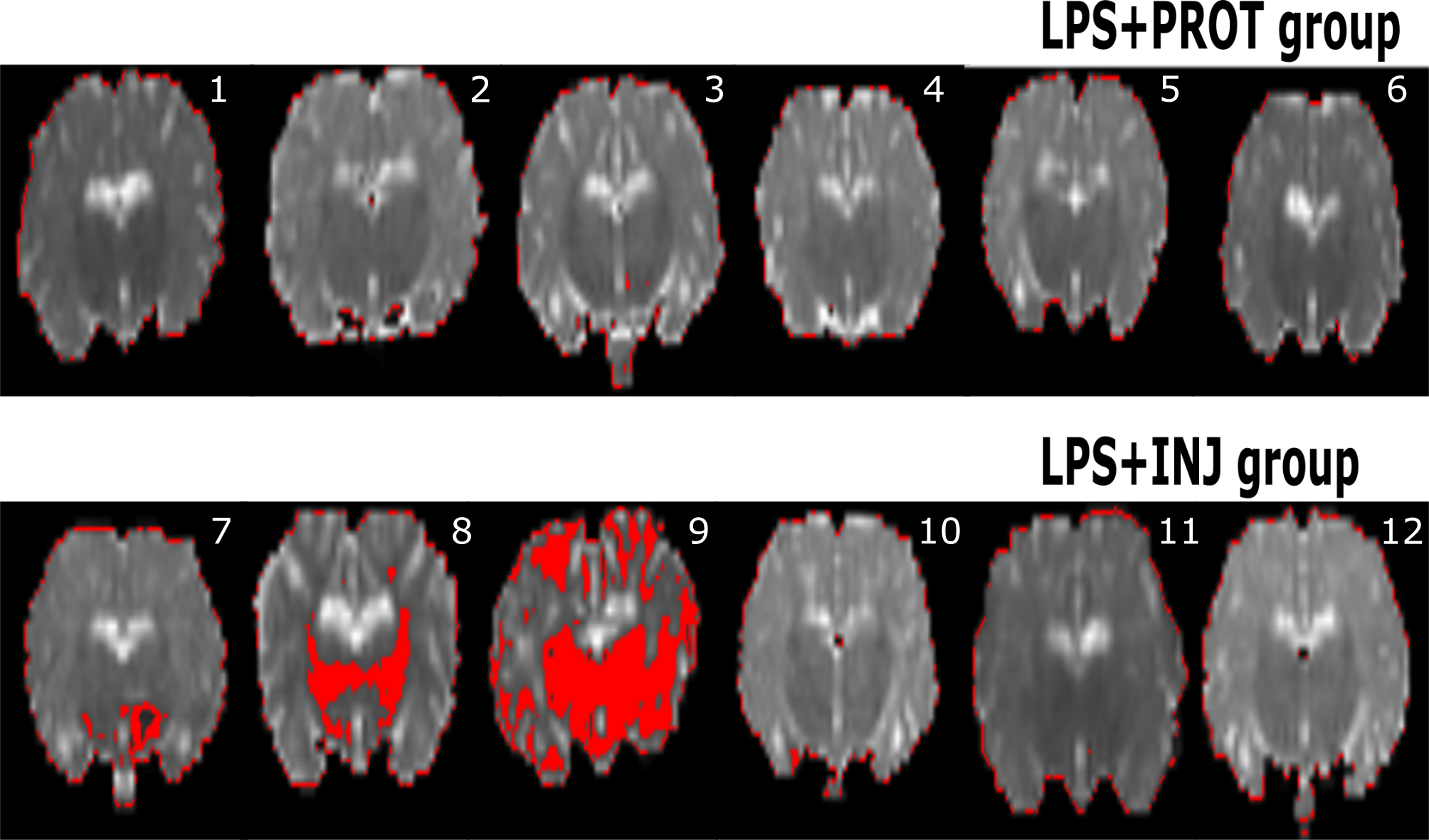

Supplement: Figure S5 — Diffusion tensor imaging-colour maps of the mean diffusivity (MD) in the thalamus; lipopolysaccharide (LPS) groups. Voxel diffusivity intensities falling below the low threshold are shown as red for MD, measurements for all lambs exposed to intrauterine inflammation (LPS). All low-diffusion maps are overlaid on diffusion images for a slice passing through the thalamus. The red colour in the images shows low MD that is more visible in the thalamus of three injured lambs (LPS + INJ) (#7–9), while the MD images of the LPS + PROT group show very few red voxels. There were reduced MD values in amygdala of one injured lamb as well (#9). Numerals in images indicate the ID for individual lambs. [file Image_5.TIF]

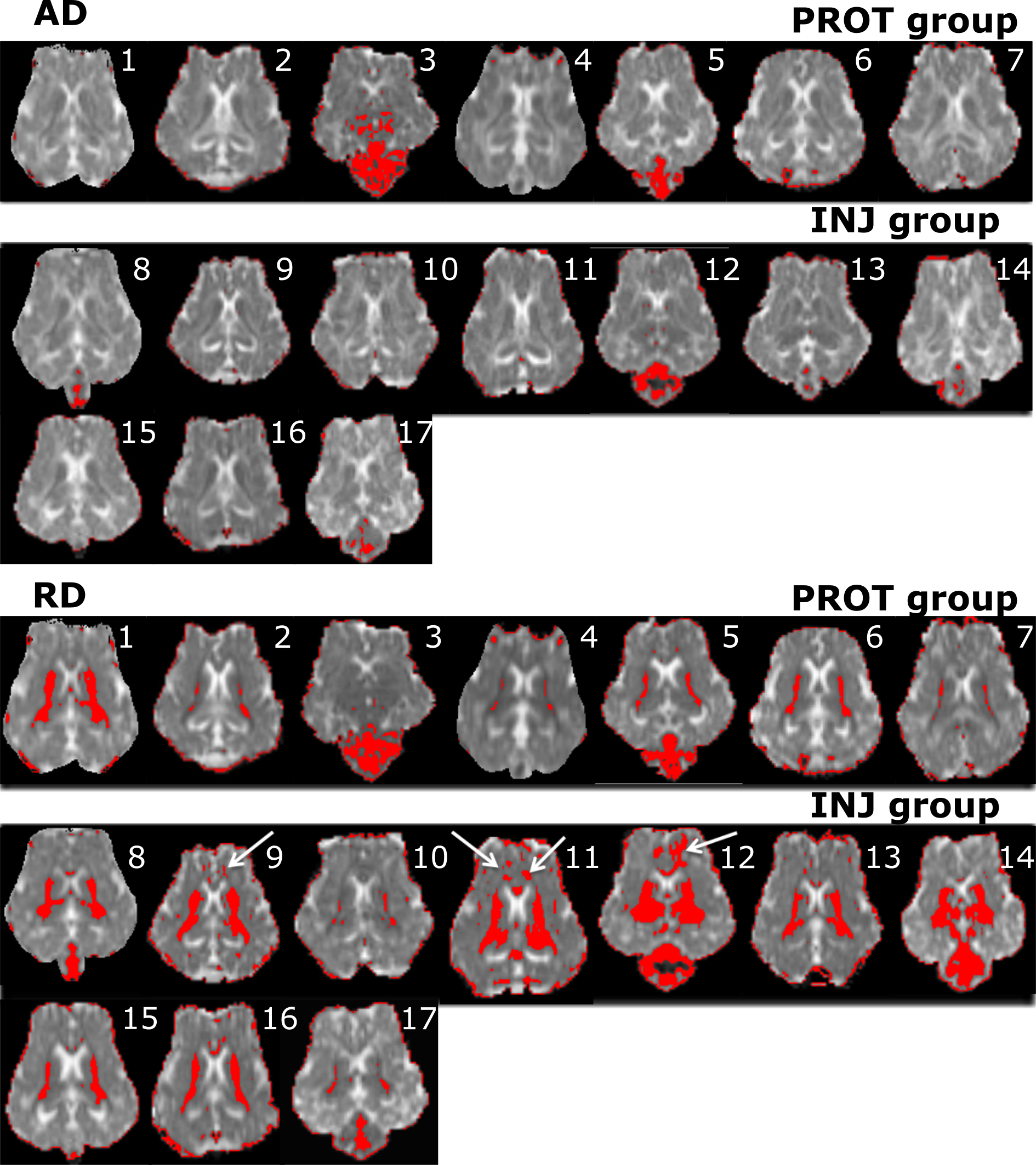

Supplement: Figure S6 — Diffusion tensor imaging-colour maps of the frontal white matter (FWM); control groups. Voxel diffusivity intensities falling below the low threshold are shown as red for axial diffusivity (AD) and radial diffusivity (RD) measurements for all control lambs not exposed to intrauterine inflammation (lipopolysaccharide). All low-diffusion maps are overlaid on diffusion images for a slice passing through the FWM. None of the lambs in either group had low AD values in the FWM, but the red colour is evident in the grey matter in some animal of both groups. In contrast, low RD values appeared in the FWM of three INJ lambs (#9, 11, and 12). Numerals in images indicate the ID for individual lambs. [file Image_6.TIF]

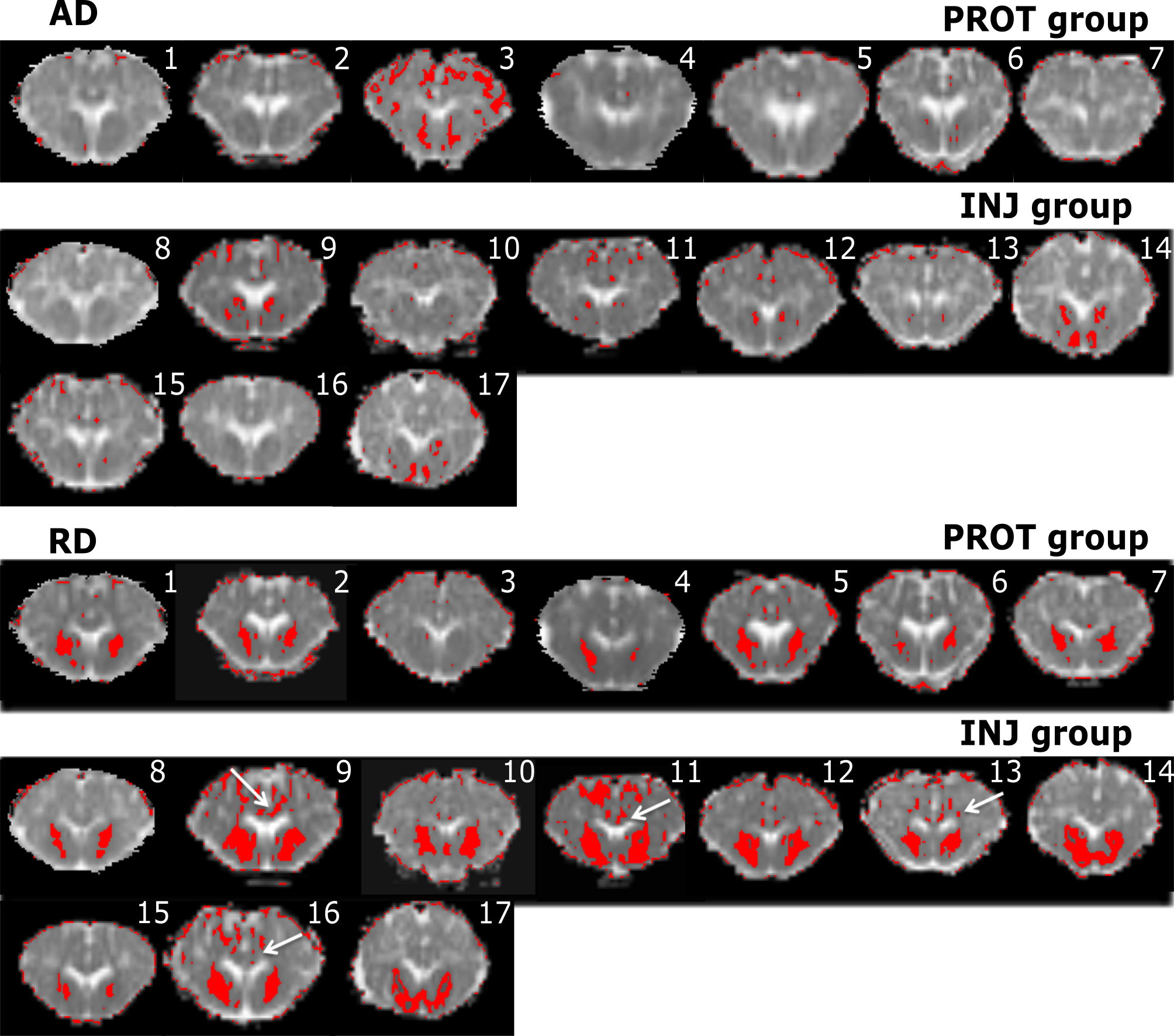

Supplement: Figure S7 — Diffusion tensor imaging-colour maps of the periventricular white matter (PVWM); control groups. Voxel diffusivity intensities falling below the low threshold are shown as red for axial diffusivity (AD) and radial diffusivity (RD) measurements for all control lambs not exposed to intrauterine inflammation (lipopolysaccharide). All low-diffusion maps are overlaid on diffusion images for a slice passing through the PVWM. There were no low AD diffusivity voxels concentrated in the PVWM in either group of lambs, but there were four INJ lambs (#9, 11, 13, and 16) with widespread low RD voxels in the PVWM when compared to PROT group lambs. Numerals in images indicate the ID for individual lambs. [file Image_7.TIF]
